# Damage Mechanisms and Anisotropy of an AA7010-T7452 Open-Die Forged Alloy: Fatigue Crack Propagation

**DOI:** 10.3390/ma15113771

**Published:** 2022-05-25

**Authors:** Tobias Strohmann, Eric Breitbarth, Michael Besel, Stefan Zaunschirm, Thomas Witulski, Guillermo Requena

**Affiliations:** 1 Institute of Materials Research, German Aerospace Center (DLR), 51147 Cologne, Germany; eric.breitbarth@dlr.de (E.B.); guillermo.requena@dlr.de (G.R.); 2 Otto Fuchs KG, 58540 Meinerzhagen, Germany; michael.besel@otto-fuchs.com (M.B.); thomas.witulski@otto-fuchs.com (T.W.); 3 FH Oberösterreich Forschungs und Entwicklungs GmbH, 4600 Wels, Austria; stefan.zaunschirm@fh-wels.at; 4 Metallic Structures and Materials Systems for Aerospace Engineering, RWTH Aachen University, 52062 Aachen, Germany

**Keywords:** fatigue crack growth, AA7010-T7452, open-die forging, microstructure–property relationship

## Abstract

The process–microstructure–property relationship of high-strength 7000 series aluminum alloys during fatigue crack propagation (FCP) is highly relevant for safety during the design and service of aircraft structural components. It is scientifically evident that many metallurgical factors affect FCP properties, but partly contradictory or inconclusive results show that the quantitative description of the relationships is still a major challenge among researchers and engineers. Most research focuses on sheet or plate products and investigations lack quantitative information on the process–property relationship between open-die forged thick products and FCP. The present study contributes to this field by investigating the fatigue crack growth behavior of an open-die forged AA7010-T7452 aluminum alloy. Four different forging conditions comprising different characteristic microstructures are comparatively analyzed. The influence of grain size, grain shape, specimen orientation, crystallographic texture, and primary phase particles is investigated. Fractographic analysis reveals different active damage mechanisms during fatigue crack growth. Based on that, the microstructure features relevant to fatigue damage areidentified in each regime of crack growth.

## 1. Introduction

The fatigue life of a material or structural component is defined by the stages of microcrack initiation, i.e., initial cyclic damage (1) followed by microcrack nucleation (2), microcrack growth (3), macroscopic crack growth (4), and instable crack growth (5) until final failure [1,2]. The first three stages (also referred to as macro-crack initiation) determine the majority of load cycles during fatigue life; however, fatigue cracks in structural components for lightweight structures such as airplanes are inherent in their fail-safe design and, therefore, the macrocrack growth and unstable crack growth define their damage tolerance [1,3,4]. Thus, inspection intervals during service life are, among others, determined based on their fatigue crack growth behavior to guarantee safety. The fatigue crack growth behavior for a given load scenario is controlled by environmental conditions and the material’s microstructure. In practice, load scenarios and environments are determined by service conditions. The microstructure, in contrast, can be modified during material processing [5]. Consequently, profound knowledge of the process–microstructure–property relationships regarding fatigue crack growth is required, e.g., for enhancing fatigue prediction tools [6].

The manufacturing process of a forged 7xxx aluminum alloy generally comprises a thermo-mechanical treatment, i.e., plastic deformation at elevated temperatures, together or followed by thermal treatment. For a given grain structure (i.e., grain morphology and crystallographic texture), the relationships between thermal treatment, precipitation kinetics, and fatigue crack propagation (FCP) were topics of intensive research and are relatively well understood [7,8,9,10,11,12,13,14,15,16]; however, even though the optimization of the precipitation condition with respect to fatigue crack growth resistance is possible, the corresponding aging kinetics affect almost all other mechanical properties, often counteractively [17].

The forming process controls the grain structure, distribution of primary phase particles, and crystallographic texture. Much effort has been put into elucidating the relationships between crystallographic texture and FCP behavior in recent years [18,19,20,21,22,23,24,25,26]. Zhai et al. proposed a crystallographic model for short fatigue crack growth at grain boundaries [18]. The twist and tilt angles of slip planes in adjacent grains of an Al-Li alloy were found to be the key factor controlling crack retardation, deflection, and branching. A high twist angle, for example, can fully stop the crack growth as the crack may not be able to enter the adjacent grain. Based on their findings, the authors derived a grain boundary geometry for optimum resistance to the growth of short cracks for Al-Li and Al-Cu alloys [19]. Other studies applied this model to longer cracks in an AA7050-T7451 thick (rolled) plate and consistently found that a growing fatigue crack can hardly enter the adjacent grain if the twist angle between the slip planes is large [20,21]. Based on these studies, Liu et al. showed that Goss grains with a low Schmid factor usually have a higher twist and tilt angle to random adjacent grains and thus have a higher resistance to FCP than cube grains and brass grains [22,23].

The impact of grain size on FCP was also investigated by several authors. Thompson and Bucci stated that grain size does not affect FCP in a polycrystalline material [27]. In contrast, other studies show grain size effects for 2xxx series aluminum alloys [28,29,30]. A higher distance between grain boundaries, i.e., barriers for dislocation movement, increases the slip distance in front of a crack. Consequently, the crack path is more tortuous, roughness-induced crack closure increases, and the crack growth rate decreases. Similar results were achieved by Kermanidis and Pantelakis. They found that a thick plate’s grain structure is “superior” to a thin sheet’s grain structure due to the coarser, elongated grains [31]. This effect might be reversed above a certain grain size [29], but a separation of the effect from other factors such as crack deflection, texture, or recrystallization is very difficult. Shan et al. investigated a three-stage homogenization reducing the fraction of small recrystallized grains by Al_3_Zr formation in AA7020. The recrystallized grain structure, therefore, yields a 30.6% faster crack growth for ΔK = 10 MPa√m [32]. Jian et al. showed the contrary effect for ΔK > 14 MPa√m, i.e., smaller grains due to sheet thickness reduction increased the FCP resistance for an L-T oriented crack [33]. Other studies investigated the orientation of the specimen with respect to the rolling direction [34,35]. Schubbe et al. showed that the FCP rate of AA7050-T7451 tested in L-S orientation is higher compared to T-L and L-T at low ΔK. This trend reverses at higher ΔK, which was associated with a tendency for retardation and splitting of the main crack in the L-S orientation, i.e., along the fiber-like grain structure parallel to the load axis [34]. Similar results were achieved by Wei et al., who ranked FCP resistances L-T > T-L > S-T at low ΔK and L-T > S-T > T-L at higher ΔK ≥ 10 MPa√m [21]. 

Brittle primary phases, e.g., Fe- or Si-containing particles, have a detrimental impact on almost all mechanical properties. Such particles easily fracture under (plastic) deformation of the Al-matrix (e.g., [36]), and, therefore, act as fatigue crack initiation sites [37] or as stress concentrations ahead of a propagating fatigue crack [33,36,38,39].

It is scientifically evident that all these metallurgical factors affect FCP behavior, while partly contradictory or inconclusive results show that the quantitative description of the underlying interrelations is still a major challenge among researchers and engineers. Moreover, most of these studies focus on thick plates [18,19,20,21,30,34,35], sheets [13,22,28,29,33], or on the comparison of both [31], which are generally produced by a rolling process. A different process to control the grain structure of 7xxx series aluminum alloys more flexibly, especially for thicker products, is forging [13,17]; however, the open literature currently lacks studies that quantitatively analyze the microstructure–property relationships during fatigue crack growth in high-strength aluminum alloy forgings.

This work provides studies on four different microstructures resulting from an industrial (i.e., full-scale) forging process, comprising a lightly deformed grain structure, a medium deformed elongated grain structure, a heavily deformed fiber-like grain structure, and a rather pancake-like grain structure owing to different forging conditions. The influence of grain size, grain shape, specimen orientation, crystallographic texture, and primary phases is investigated. Firstly, we show the different grain structures and textures as well as the corresponding FCP behavior. Secondly, we identify the active damage mechanisms during fatigue crack growth. Based on that, the dominating microstructural features are identified in each crack growth regime and are then correlated to the fatigue crack growth behavior.

## 2. Materials and Methods

### 2.1. Material

The material investigated was an open-die forged high-strength AA7010-T7452 alloy with the nominal composition given in Table 1.

Zirconium was added to promote the formation of Al_3_Zr dispersoids to improve the recrystallization resistance [40]. This ensures that different forging routes yield different microstructures with grain morphology (size, shape) and crystallographic texture clearly reflecting the history of plastic deformation during forging.

The processing and heat treatment routes consisted of the following:

1. Casting a cylindrical forging stock;
2. Multiple forging steps to achieve the final plate-like shape of the blanks; forging temperature ~400 °C;
3. Solution heat treatment at 475 °C for 4.5 h;
4. Water quenching;
5. Cold deformation/cold heading to relieve residual stresses;
6. Overaging in two steps: 120 °C/20 h followed by 175 °C/7 h.

All specimens were taken near the center of the open-die forged plates. Four forging variants were achieved depending on the dimensions of the forged bar, its orientation during forging, and the shape of the final blank.

For variants 1 to 3 (var 1–3) the major axis of the cylindrical forging stock (its casting direction) was parallel to the forgings’ final T direction, whereas in the case of var 4 it was parallel to the S direction. Variants 1 to 3 can be interpreted as three states during an ongoing forging process, where the forging blank is step-wise elongated. In this context, variant 1 has the lowest deformation state, i.e., its microstructure reflects the beginning of a forging process. Var 2 comprises a state of medium deformation, whereas var 3 experienced the highest accumulated plastic deformation. A simple but straightforward estimation of the resulting grain shapes assuming nearly equiaxed grains in the casting/forging stock and elongated grains after forging gives the aspect ratios L-T-S shown in Table 2. For this calculation, the dimensions of the cylindrical forging stock are simply compared with the outer dimensions of the final forging. Since no pronounced recrystallization occurs and continuous, locally homogeneous plastic flow prevails (especially close to the center), the outer dimensional changes achieved during forging qualitatively reflect the average shape of the elongated grains.

### 2.2. Fatigue Crack Growth Experiments

**Figure 1:**
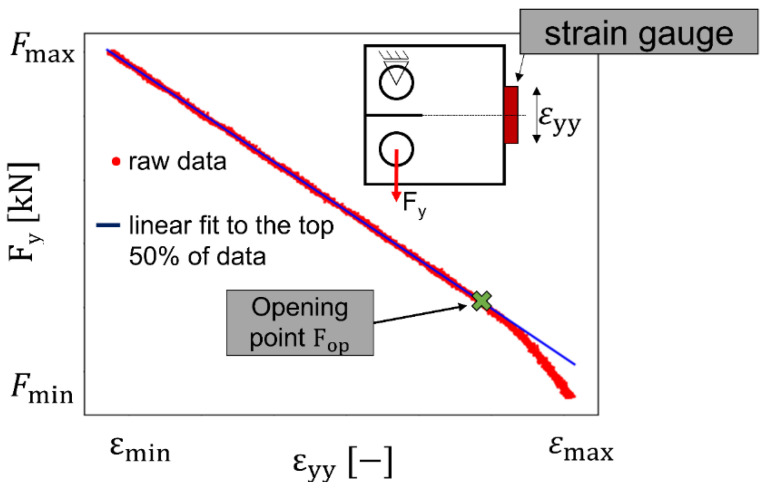
Exemplary F_y_ vs. ε_yy_ curve (ε_yy_ is measured by a strain gauge at the specimen’s backside). The linear interpolation (blue) shows the material response for a fully open crack.

Fatigue crack propagation experiments were performed for the orientations L-T and T-L (i.e., loading in L and FCP in T, and loading in T and FCP in L directions, respectively). The tests were carried out in a standard uniaxial servo-hydraulic testing rig with a maximum load capacity of 60 kN controlled by the Instron software “Advanced Crack Growth Application Software Program”. Standard CT75 specimens (width W = 75 mm) with a thickness t = 12 mm and an initial 24 mm pre-crack from an 18.75 mm V-shaped starter notch were investigated [41]. A constant cyclic load amplitude ∆F = 2.7 kN with a load ratio R = 0.1 was applied for the rising ∆K method, following the standard ASTM E647 [42]. The initial ∆K for the crack growth experiments was 5 MPa√m. The crack length was measured by means of direct current potential drop utilizing Johnson’s formula [43]. The experiments were performed at room temperature and standard laboratory conditions applying a 20 Hz sinusoidal waveform. Data were acquired for every 0.1 mm of crack extension. The FCP rate da/dN was calculated using the Increment Polynomial Method [42]. Furthermore, the crack opening force was determined according to the following procedure:

1. The strain and force signals were both measured with an HBM precision measuring amplifier to keep the phase shift between both signals low. In addition, the remaining phase shift was corrected by a Peak-To-Peak fit to synchronize the measurements. The strain gauge was located at the specimen’s back face (see insert in Figure 1). The strain signal was recorded every 0.2 mm increment of crack extension at a sampling rate of 9.6 kHz for a time interval of 0.5 s, i.e., each set of load-strain data contains 10 load cycles.
2. A resulting F_y_-ε_yy_ curve over these 10 load cycles is exemplarily shown in Figure 1. The graph shows a decrease in the slope of the F_y_-ε_yy_ curve as the strain decreases (i.e., higher compression of the specimen’s back face) until a linear region is reached. The point at which this linear dependence starts indicates a fully open crack and therefore corresponds to the crack opening force F_op_ (Figure 1). F_op_ can be used to obtain the opening stress intensity factor K_op_.
3. The cyclic stress intensity factor (SIF) ΔK was corrected using the opening forces as ΔK_eff_ = K_max_ − K_op_.

### 2.3. Metallography and Quantitative Fractography

Scanning electron microscopy (SEM) was performed using a Zeiss Ultra 55 FEG device equipped with an Oxford INCA EDX system and the Oxford Nordlys II EBSD system. The acceleration voltage was set as 15 kV for SEM and 20 kV for electron backscattered diffraction (EBSD) scans. Images by type II secondary electrons (SE2) reveal topology information of the fracture surfaces, while backscattered electrons (BSE) images are linked to the compositional contrast.

Fracture surfaces were cut from the CT75 specimens and cleaned with compressed air followed by ultrasonic cleaning in ethanol and distilled water. The base material was ground and polished by standard metallographic procedures, including grinding, diamond paste, and SiC-based polishing agent.

The determination of FCP mechanisms and their quantification was carried out by analyzing SEM images of all specimens along a crack length a = 30–50 mm (∆K = 6–15 MPa√m). The following procedure was carried out to identify the dominant damage mechanisms:

1. The fracture surface was imaged by SEM over a region of 0.6 × 20.0 mm².
2. SEM images of an area ~117 × 117 µm² were selected randomly without knowledge of the location. This was performed to avoid human biasing during imaging analysis.
3. Each of these micrographs was classified manually according to the active damage mechanisms.
4. Finally, all images were stitched together and the area fraction for each damage mechanism was quantified as a function of the crack length.

Specimens for EBSD analysis were cut from the corner of the tested CT75 specimens. Samples were ground using #500–#4000 grinding paper and electropolished in a solution of methanol and 40% nitric acid (HNO3) in a relation of 4: 1 at −12 °C to −14 °C acid temperature. The EBSD measurements were carried out using the software Aztec (v. 3.x–4.x) by Oxford Instruments (High Wycombe, UK) and analyzed in Oxford Instruments Channel 5 (v. 5.05) software. The beam’s step size was set as 1.25 µm in each scan. Grain dimensions in the transverse direction were calculated by the line intercept method based on the angular differences of orientations measured by EBSD. One hundred lines were placed parallel to the T direction in the L-T plane and T-S plane. Grain boundaries were identified by a predefined orientation difference angle ≥ 15° between adjacent grains. A minimum distance of 3.75 µm (3 pixels) between two adjacent grain boundaries was chosen to define a new grain. It is important to point out that the distance between grain boundaries is at least one order of magnitude larger than the step size of 1.25 µm used for the EBSD scans.

A weighted distance between the high angle grain boundaries is used for a quantitative discussion of the results: A histogram from the conventional (i.e., not-weighted) line interceptions over-represents small grains due to their higher count; however, depending on the crack configuration with respect to the spatial arrangement of large and small grains, the effects of the larger grains dominate the FCP as the crack more often propagates through larger grains. To overcome this issue, each grain boundary distance was counted equally to its length, i.e., a 15 µm grain is counted 15 times and a 100 µm grain is counted 100 times. In principle (assuming spatial independence in grain size), this procedure reflects the probability of a crack tip at a random position being in a grain of a specific size.

### 2.4. X-ray Computed Tomography

X-ray computed tomography (XCT) was performed at the University of Applied Sciences Upper Austria, on an RX Solutions Easytom 160, equipped with a 160 kV Hamamatsu X-ray tube and a 1920 × 1536 pixels flat panel detector from Varian. The acceleration voltage was set to 70 kV with 108 µA using a LaB6 filament, middle focus, and diamond/tungsten target. The voxel size was set to (1.3 µm)³ with detector shift for the reduction in ring artifacts and an average of 6 images for each projection of the 1440 images. To cover a higher volume along the T-direction, multiple scans were carried out. A 3D analysis was performed using VG Studio 2.2 MAX (Volume Graphics GmbH, Heidelberg, Germany) and Aviso 9.5.0 (Thermo Fisher Scientific, Waltham, USA). The data were filtered by means of a non-local mean filter to enhance the contrast of edges while smoothing the background noise. The surface of the primary mode I crack was eroded for 4 pixels in the L-T slices and the segmentation of particles and secondary cracks followed a standard global grey value threshold scheme plus additional manual segmentation.

## 3. Results

### 3.1. Grain Structure and Crystallographic Texture

**Figure 2:**
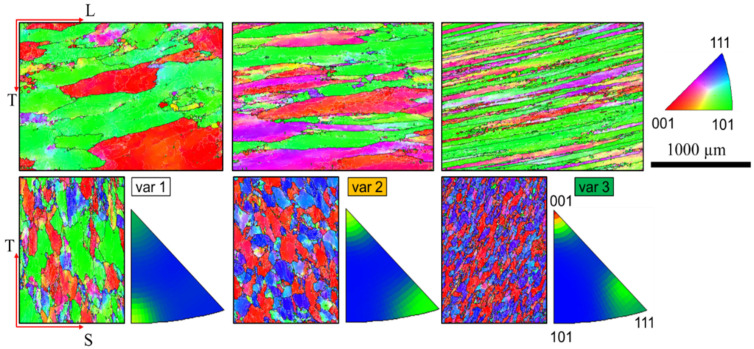
Inverse pole figure EBSD mapping showing the grain structure with high angle grain boundaries > 15° for the AA7010-T7452 var 1 to var 3, respectively. The colors indicate the crystallographic direction normal to the material’s plane shown, i.e., the colors for the L-T-plane (top row) show the crystallographic direction parallel to S. Readers are highly recommended to read the pdf version of the article for color visualizations.

Figure 2 shows an inverse pole figure (IPF) obtained by EBSD, representing the crystallographic orientation of var 1 to var 3. The grain structure is continuously elongated with the ongoing forging process from var 1 to var 3, respectively. This is best visible on the L-T plane, as grains become thinner in the T direction while a fiber-like structure with elongated grains in the L direction evolves. Moreover, the T-S grains’ profiles progress from slightly elongated in the T direction to a more globular appearance, from var 1 to var 3, respectively. Furthermore, the projected grain area in the T-S plane decreases. The evolution of the grain structure is complemented by a sharpening of the crystallographic texture. The crystallographic direction pointing in L-direction (IPF colors in T-S plane) is blurred between the [101] and directions (var 1). The crystals then rotate, and a more pronounced texture develops with [001] and [111] pointing in the L direction. This behavior is intensified from var 2 to var 3. Consequently, the [001] and [111] directions are precisely pointing in the L direction.

**Figure 3:**
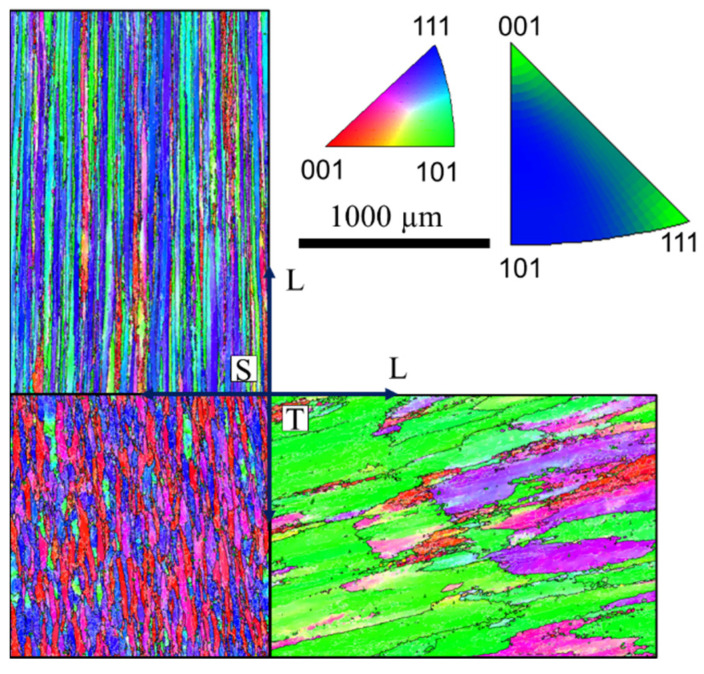
EBSD map showing the grain structure with high angle grain boundaries > 15° for the AA7010-T7452 var 4. The colors indicate the crystallographic direction normal to the material’s plane shown, i.e., The colors for the L-T-plane show the crystallographic direction parallel to S.

Figure 3 shows the EBSD IPF and grain structure for var 4, i.e., a modified casting direction, parallel to S. The change of the casting direction yields a highly elongated grain structure (see Table 2); however, grains are also elongated in the T direction while their S direction becomes very short. Consequently, the L-S aspect ratio is extremely high, although the L-T aspect ratio is similar to var 2. The evolution of the grain structure is again accompanied by a texture evolution. This is similar to var 2, however less clear, with [111] and [001] pointing in the L direction.

**Figure 4:**
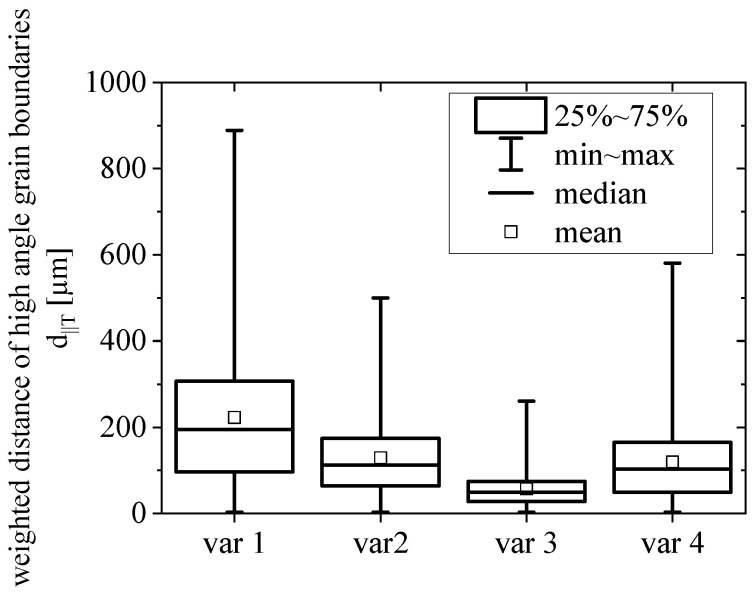
Distance between high angle grain boundaries (>15°) in T direction. The box plot was achieved via a weighting procedure taking the grain sizes into account.

Figure 4 compares the (weighted) high angle (>15°) grain boundary distances parallel to T direction d_||T_ for the different variants. The largest grains (with respect to T direction) are found in var 1, as the grain structure is not yet fiber-like (see Figure 2). The grain boundaries show distances of up to 900 µm; however, the mean distance in the T direction is ~220 µm. The grain boundary distance in the T direction consistently decreases from var 1 to var 3, i.e., with an extended forging procedure, while var 4 shows a T direction grain distance similar to var 2.

**Figure 5:**
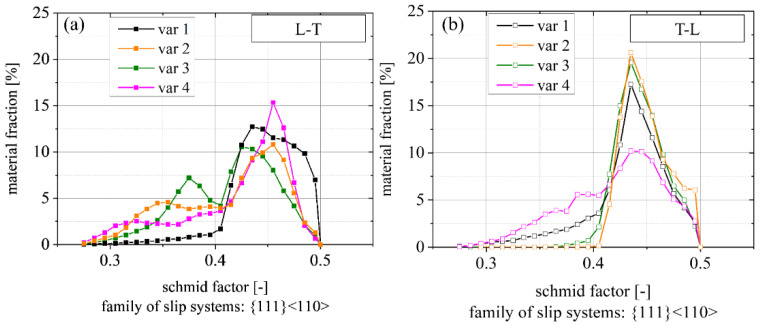
Schmid factors for {111} <110> family for load direction parallel to (**a**) L and (**b**) T directions.

Figure 5 shows the distribution of the Schmid factor m calculated from the EBSD analysis. The Schmid factor is shown for the {111} <110> slip system family for L-T (loading direction parallel to L) and T-L (loading direction parallel to T), respectively. In both directions, one peak is present at a Schmid factor m ≈ 0.43. For var 1 to 3, this peak is clearly pronounced in the T-L orientation, while var 4 shows a pronounced peak in L-T orientation. Overall, more crystals with a lower Schmid factor, thus, lower resolved shear strength, are present for the load parallel to the L direction, i.e., L-T orientation. The fraction of these grains increases from var 1 to var 2 and var 3. The lower Schmid factor is mainly associated with a Goss texture, i.e., with the {111} plane parallel to the T-S plane and [112] direction normal to the L-T plane (see Figure 2).

### 3.2. Fatigue Crack Propagation

**Figure 6:**
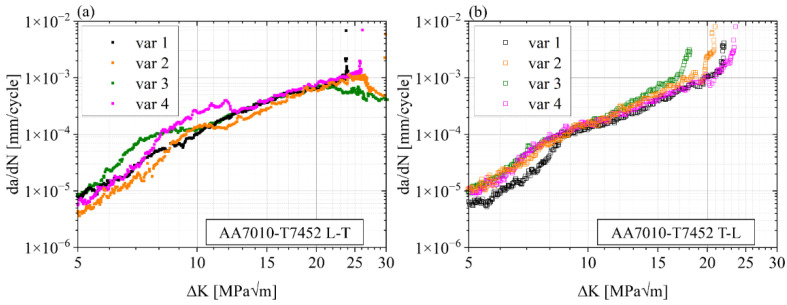
AA7010-T7452 crack growth curves da/dN-ΔK for L-T (**a**) and T-L (**b**) orientations.

The crack extension per cycle da/dN is shown as a function of the cyclic SIF ΔK in Figure 6. Especially the L-T orientation (a) shows large differences between the four variants for ΔK ≤ 13 MPa√m. In the range between 13 MPa√m and ΔK ≤ 22 MPa√m, crack growth rates align with only minor differences until the specimens reach their unstable FCP regimes. Starting around 22 MPa√m and 26 MPa√m (var 3 and var 2, respectively), secondary cracking occurs in var 2 and var 3. Consequently, the computed theoretical stress intensity factors are higher than the actual ΔK, and thus, FCP rates decrease. This effect is discussed in detail in Section 4.4. In the case of T-L orientations, the da/dN-curves are relatively close together. Overall, var 1 shows the lowest FCP rate. For ΔK ≥ 11 MPa√m, var 2 and var 3 undergo a faster crack growth than var 1 and var 4. This behavior is equivalent in the region of final failure.

**Figure 7:**
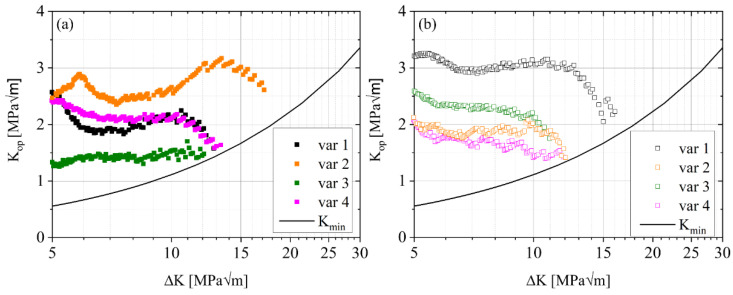
Opening stress intensity factor K_op_ determined by the measured crack opening force F_op._ (**a**) in L-T orientation and (**b**) in T-L orientation.

Crack opening forces and opening stress intensity factors were calculated during the fatigue crack propagation tests using a back-face strain gauge following the post-processing described in the methodology section. The resulting K_op_ is shown in Figure 7. Large differences (K_op_ ≈ 1.5–3 MPa√m) are observed between the different variants as well as between the L-T (a) and T-L (b) orientations. In most cases, the opening stress intensity factor shows only minor variations in a range less than ±0.5 MPa√m before dropping to values close to the nominal K_min_, i.e., the closure effect disappears.

**Figure 8:**
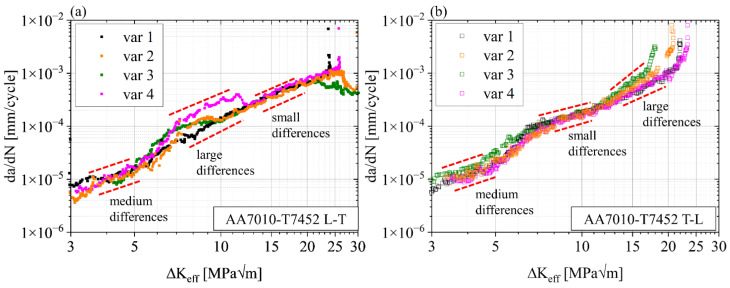
∆K_eff_ fatigue crack propagation curves of AA7010-T7452 in four open-die forged conditions for (**a**) L-T orientation and (**b**) T-L orientation.

The opening stress intensity factors were used to calculate the effective cyclic stress intensity factors by Elber’s approach, i.e., ΔK_eff_ = K_max_ − K_op_ [44]. The resulting da/dN − ΔK_eff_ curves are shown in Figure 8 for L-T (a) and T-L (b) orientations, respectively. The FCP rates of the L-T orientations of the different variants show a high scatter band for cyclic SIFs ΔK_eff_ ≤ 5.5 MPa√m while partially maintaining the individual characteristics between 5.5 MPa√m < ΔK_eff_ ≤ 12 MPa√m. Beyond this region, the FCP rates align even more compared to the original plot (Figure 6) due to the correction of var 2 data (i.e., the only variant with active crack closure beyond this point). T-L data of the different variants share a comparably small scatter band where var 3 with the strongly elongated grains (fiber-like) shows slightly higher FCP rates up to ΔK_eff_ = 6 MPa√m. The data for variants 2–4 in the region of ΔK_eff_ > 12 MPa√m are identical to the original plot in Figure 6, as their crack closure effects disappear around ΔK ≈ 12 MPa√m (see Figure 7).

As all data in Figure 8 are separated from extrinsic crack closure effects, the specific features of these curves may be directly attributed to intrinsic effects caused by the microstructure ahead of the propagating crack tip.

### 3.3. Damage Mechanisms

**Figure 9:**
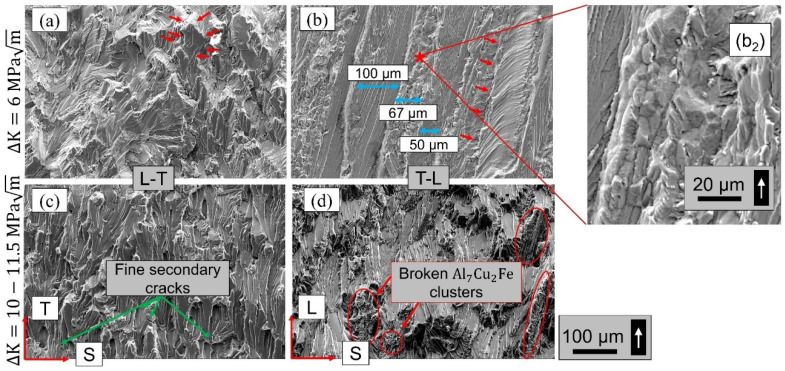
SEM micrographs of fracture surfaces of var 3 (**a**), (**c**) L-T and (**b**), (**d**) T-L samples of low ΔK (**top row**) and higher ΔK (**bottom row**). The white arrow indicates the crack propagation direction (**bottom to top**). (**b_2_**) Inter-(sub-) granular fractured region of (**b**) at higher magnification.

The evolution of damage mechanisms is representatively discussed for var 3 in Figure 9. The fracture surface is shown at low (top row, ΔK ≈ 6 MPa√m) and higher ΔK (bottom row, ΔK ≈ 10–11.5 MPa√m) for L-T (a, c) and T-L (b, d), respectively. The fracture surfaces at low ΔK represent the grain structure in the respective T-S and L-S planes (compare Figure 2), parallel to the fracture surfaces. In the L-T orientation, the crack grew mostly trans-granularly and changed its local direction when bypassing grain boundaries (red arrows in Figure 9a). Consequently, a homogenous, globular, facet-like morphology is present for the L-T orientation. In T-L orientation, a fiber-like morphology is observed. These fibers-like grains are 50–100 µm thick, and represent the larger grains of var 3 (compare Figure 4). Inter-(sub-) granular fractured regions can be found between the trans-granular regions, as pointed out in the inset (b_2_). For higher stress intensity factors, the fracture morphology of both orientations change (sub-images c and d). Plateaus are revealed, which show striations at higher magnification (not shown here). Very fine secondary cracks are present in the L-T orientation. Broken clusters of primary Al_7_Cu_2_Fe can be observed on the T-L fracture surface.

**Figure 10:**
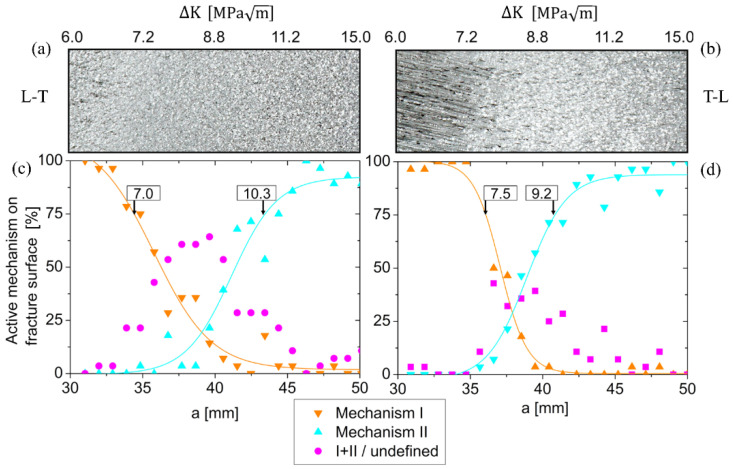
Quantitative analysis of damage mechanisms on the fracture surface of var 3 L-T (**a**,**c**) and T-L (**b**,**d**) orientations. Macrographs from the optical microscope of the fracture surfaces are shown at the top.

The var 3 fracture surfaces were analyzed quantitatively following the description in the methodology section. The result is shown in Figure 10. Mechanism I refer to a locally more crystallographic-based fracture process resulting in the clear appearance of the underlying grain structure. Mechanism II refers to smaller plateaus with striations indicating large-scale activation of alternating slip ahead of the crack tip. In both orientations, L-T and T-L, mechanism I dominates fracture in the early part of the experiment: In both orientations, 75–100% of the fracture surface is covered by mechanism I up to ΔK > 7.0 MPa√m (L-T), and 7.5 MPa√m (T-L), respectively (this corresponds to crack length of 35–40 mm). Then, a region appears where neither mechanisms I nor II clearly dominate fracture. Instead, a mixture of both can be found, indicating a transition regime during further crack growth. Finally, mechanism II becomes dominant at higher stress intensity factors > 9.2–10.3 MPa√m. In the case of T-L, this change in the damage mechanism is easily seen even on a macroscopic level (see macrograph in sub-image b) because visual differences between fiber-like facets (mechanism I) and much finer plateaus (mechanism II) are very large.

## 4. Discussion

In the following, we discuss the results obtained from eight crack growth experiments, i.e., four open-die forged conditions in L-T and four in T-L orientation. A limitation of fatigue crack growth studies is their relatively low reproducibility [42]. Due to the fact that FCP experiments are expensive and time-consuming [2], the repetition of experiments for each condition is often not feasible. We tackle this limitation with an integrated approach aiming at the identification and validation of (small) differences in the FCP data combining the experimental results with a detailed analysis of underlying mechanisms at the microstructural level.

### 4.1. Damage Mechanisms and their Transition Regime A_3_

**Figure 11:**
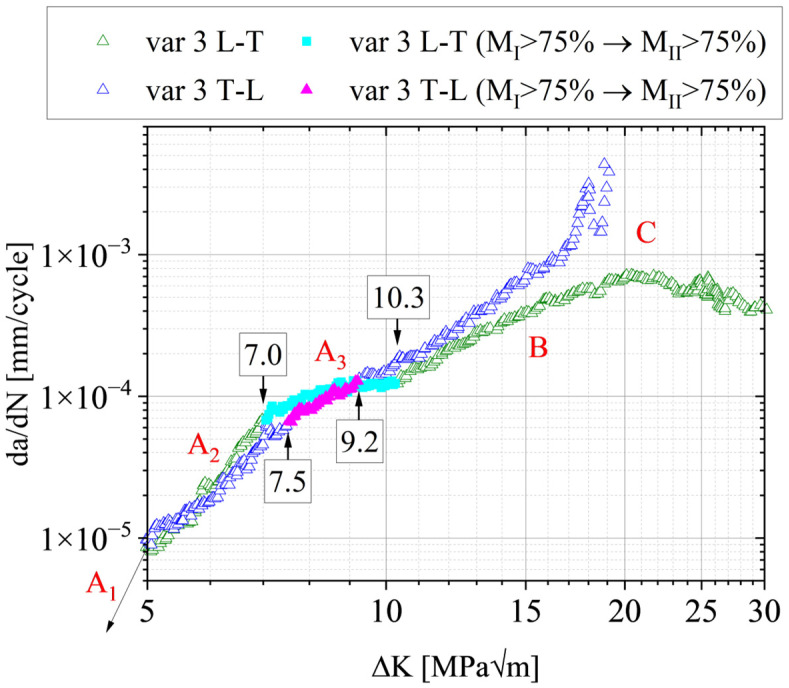
AA7010-T7452 FCP data of var 3; highlighting transition of damage mechanism. The regime begins when the fraction of damage mechanism M_I_ is smaller than 75% and ends when the area fraction of mechanism M_II_ is greater than 75%. Regime A_1_, usually called the threshold regime, was not part of the study.

The correlation between da/dN-ΔK and the change of the damage mechanism are presented exemplarily for var 3 in Figure 11. The regime where neither mechanisms I nor II are dominant, i.e., both <75%, are highlighted. We call this regime the transition regime. It becomes clear that the transition between mechanisms I and II correlates well with the slope change in the mid-section of the curves. The fact that such a correlation exists is not new—the graph combined with Figure 9 and Figure 10 quantifies the transition in a very detailed way. These results state that a transition *point,* as was usually suggested by other works (e.g., [45,46] or [47]), does not exist. Instead, a transition *regime* is present; see A_3_ in Figure 11. Such a transition regime is well aligned with other studies, which show that damage mechanisms during FCP usually change gradually [28,48].

The preceding regime of A_3_ is regime A_2_. We refer to the mechanistic meaning of the crack growth regimes for the nomenclature, i.e., regime A is controlled by the microstructure and the crack grows quasi-individually in different grains, i.e., direction and propagation rate may change from grain to grain depending on their mismatch of slip plane orientations. In contrast, regime B is insensitive to microstructure and is log–log linear. Regime C is the regime of final fracture [49].

**Figure 12:**
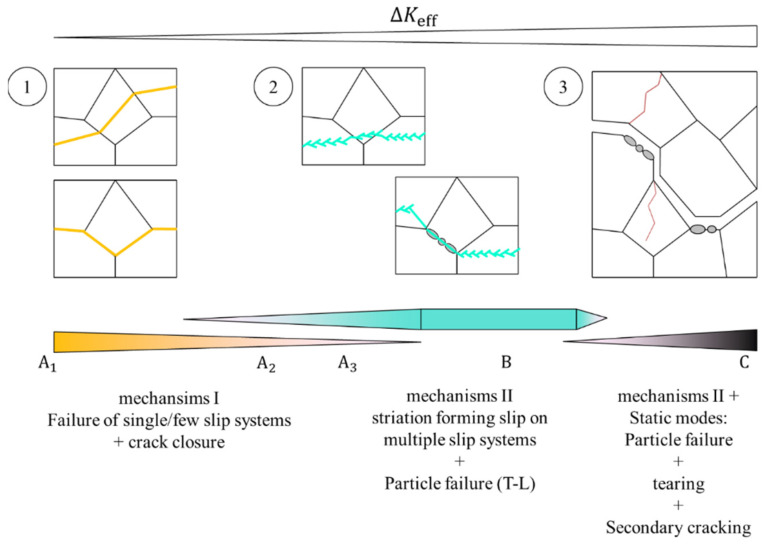
Schematic representation of the evolution of damage mechanisms as a function of ΔK.

Figure 12 schematically summarizes the evolution of the damage mechanisms. Mechanism I dominates the fracture during regime A. The plastic zone size is small compared to microstructural features (e.g., grain-/sub-grainsize); the crack grows individually from grain to grain, changing its direction and growth rate, and significant crack closure effects are observed. The active damage mechanisms change gradually in the transition regime. Mechanism II becomes dominant, initiating a comparably microstructure-insensitive, striation-forming crack growth. At this stage, clusters of broken Al_7_Cu_2_Fe particles are found on the fracture surface of T-L specimens, indicating that Al_7_Cu_2_Fe particle fracture becomes more relevant. Mechanism II is accompanied by static modes such as tearing and frequent particle fracture in regime C. Moreover, secondary cracks orientated mainly parallel to the loading direction become relevant, as pointed out in the last part of the discussion.

This evolution of damage mechanisms is qualitatively independent from the forging variant as it is found in all four variants; however, the exact transitions and the resulting crack growth rates in each regime are highly dependent on the grain structure and texture, which are both controlled by the forging process. For each regime (we do not discuss the threshold regime A_1_ due to the lack of experimental data for ΔK < 5 MPa√m), the details of relevant microstructure features are discussed separately in the following section.

### 4.2. Regime A_2_

**Figure 13:**
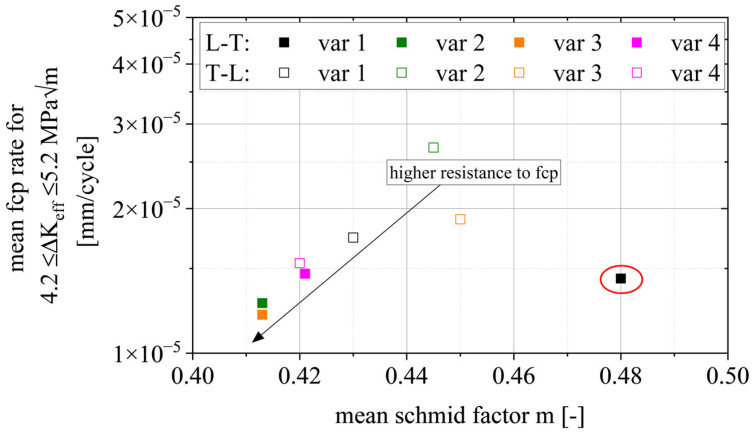
Correlation of mean crack growth rate da/dN in region A_2_ and mean Schmid factor m for {111} <110> slip system family with respect to the load direction.

Cracks in face-centered cubic (FCC) crystal systems preferentially grow along slip planes of the closed packed {111} plane family where (compared to other slip planes) lower energy is necessary to move dislocations in front of the crack tip; however, other studies have shown that crack growth along {110} or {100} planes is also possible [50]. The Schmid factor, as an indicator for the resolved shear stress in a specific slip system, can also be interpreted as a relative measure of the energy available to activate this slip system. Figure 13 shows the correlation between the mean FCP rate in the early stage A_2_, i.e., between ΔK_eff_ = 4.2–5.2 MPa√m and the mean Schmid factor m (see Figure 5). A higher Schmid factor consistently coincides in most cases (outlier for var 1 L-T) with a higher crack growth rate. In detail, an increased Schmid factor from m = 0.413 to m = 0.445 comes with a ~60% higher mean FCP rate. The outlier var 1 L-T may be caused by different reasons. One is the comparably large grain size with respect to the size of the EBSD-scanned region, i.e., the scanned region is not representative. Another reason may be the large grain size itself compared to plastic zone size and possible substructures that might not be captured during analysis.

To summarize, except for var 1 (L-T), the Schmid factor is a good indicator for crack growth rates in the lower ΔK regime, i.e., when crack growth is crystallographic-based mainly by slip plane cracking; however, other factors, especially twist and tilt angle between adjacent grains [20,21] and the ratio of specific crystallographic orientations and random orientations [22] are also relevant and interact with each other.

### 4.3. Regime B

**Figure 14:**
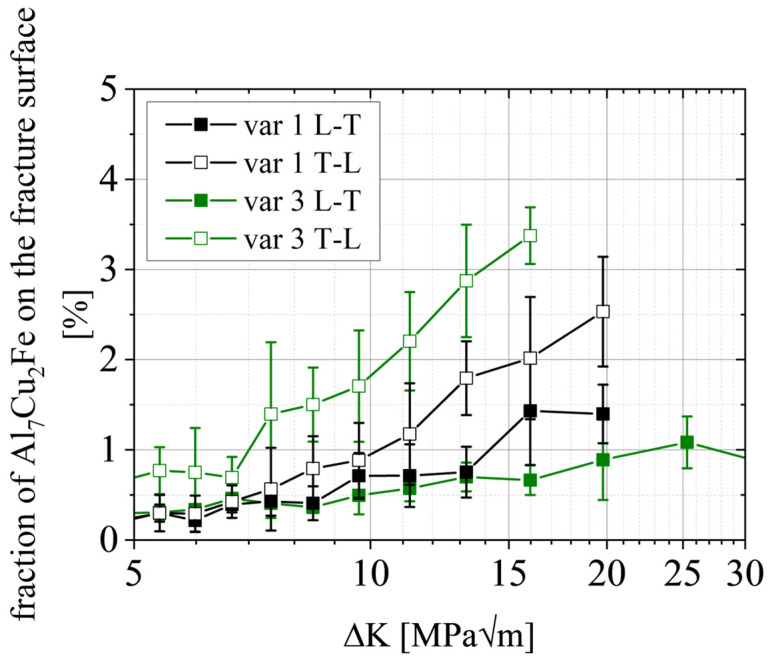
Area fraction of Al_7_Cu_2_Fe-type particles on the fracture surface as a function of ΔK for forging variants 1 and 3, respectively.

Once the damage mechanism changes into the striation phase, the crack growth rates in the L-T orientation are similar for all variants (see Figure 8a). On the other hand, acceleration is observed for T-L. The fracture surfaces show that more Al_7_Cu_2_Fe particles are involved in the fracture process, especially for the T-L orientation, because of clusters distributed along the L direction and therefore in the primary crack growth direction. The influence of coarse particles can be expected to rise with the length of these clusters parallel to the crack growth direction. The particles are very brittle and weak against plastic deformation of the surrounding matrix [36]. Consequently, they break already before the crack actually reaches the particle cluster, especially for higher ΔK or K_max_ [38,39]. This causes a “jump” of the crack once it reaches a broken cluster and the crack is locally accelerated [33]. The fraction of Al_7_Cu_2_Fe on the fracture surface is therefore expected to rise with the L:T aspect ratio for the T-L orientation and decrease with the L:T aspect ratio for the L-T orientation. This behavior is evidenced in Figure 14 for var 1 and var 3, respectively. For all curves, the fraction of the primary phase on the fracture surface increases as a function of ΔK; however, the level of increase is very different. The L-T oriented specimens show only a slight increase up to an area of 1–1.5% of the fracture surface. In contrast, the T-L orientation shows a larger increase up to 2.5% (var 1 T-L) and 3.5% of the fracture surface (var 3 T-L).

**Figure 15:**
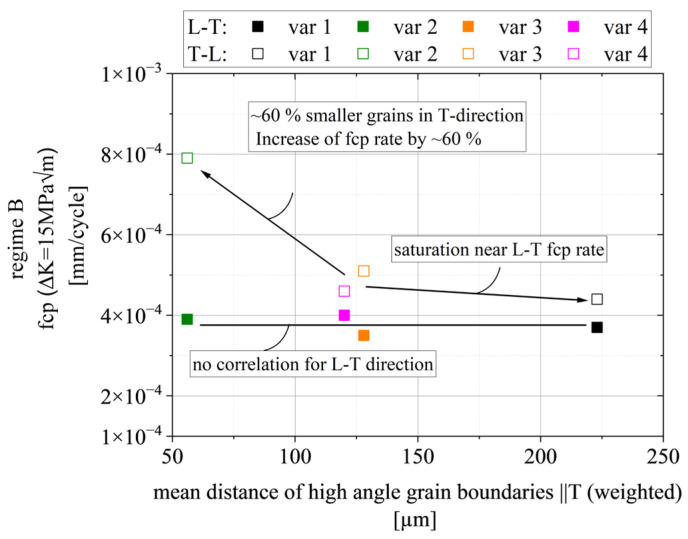
Correlation of the crack growth rate in regime B and the mean weighted distance of high angle grain boundaries in the T direction.

To quantify the correlation more in detail, Figure 15 presents the FCP rate at a stress intensity factor ΔK = ΔK_eff_ = 15 MPa√m as a function of the mean distance of high angle grain boundaries parallel to the T direction (see Figure 4). The grain size in the T direction is equivalent to the distance of particle clusters and therefore indicates the probability that a crack finds a particle cluster ahead. Moreover, grain boundaries are low cohesion zones and therefore weaker crack paths. The graph shows no correlation for the L-T orientation since the fiber-like grains are perpendicular to the primary crack growth direction, thus, not affecting the crack growth rate. On the other hand, the smaller grains (with respect to the T direction) in var 3 (~60% smaller) increase the FCP rate by ~60% in the T-L orientation; however, larger grain boundary distances in the T direction increase the FCP resistance in region B only up to a level of saturation, which is similar to the case in the L-T orientations. We conclude a microstructure-insensitive crack growth for T-L specimens once this saturation is reached.

From the above analysis, we derive the log–log–linear Paris relationship da/dN = 2.5 × 10^−7^ ΔK_eff_^2.7^ for all L-T orientations equally. One can increase the slope for T-L by a factor that is a function of the mean distance of high angle grain boundaries in the T direction.

### 4.4. Regime C

**Figure 16:**
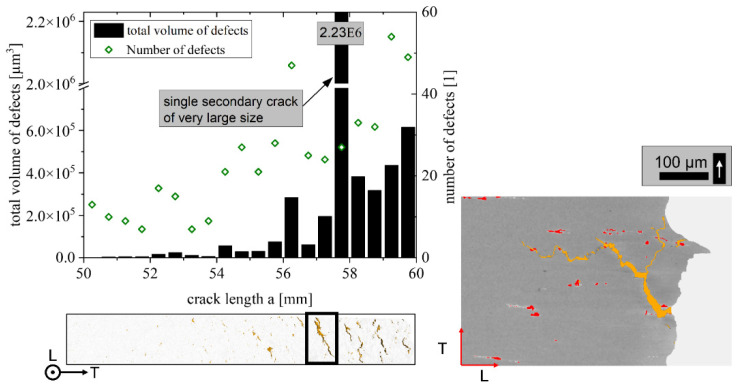
Number and volume of secondary cracks and defects at high crack lengths for var 3.

Regime C is also dominated by particle fracture and tearing along high-angle grain boundaries. Consequently, the grain boundary distance in the T direction is again the most relevant parameter. Moreover, defects evolve perpendicularly to the main crack’s direction, which yields a lower effective driving force of the main crack tip because of energy distribution between both crack tips. Figure 16 shows the number of defects in var 3 L-T summed up for every 0.5 mm crack growth and ~1 mm thickness. The volume of defects, as well as the number of defects, increase rapidly for > 55 mm, promoting failure of the specimen perpendicular to the mode I crack propagation; however, this happens only for var 2 and var 3, as the grain size in T direction is low, the L direction is very elongated and, additionally, the T:S aspect ratio provides relatively flat paths along the grain boundaries in the L-S plane, i.e., the T:S aspect ratio is not too high. The findings are in good agreement with other studies that show the tendency of splitting and branching of the primary crack at high ΔK along the longitudinal direction perpendicular to the primary crack [21,34].

## 5. Conclusions

We investigated the fatigue crack growth behavior of an industrially scaled open-die forged high-strength AA7010-T7452 aluminum alloy. The novelty of our study is a detailed and quantified investigation of the correlation between forging conditions, grain structure, primary intermetallic particles, and fatigue crack propagation; therefore, the study is a step towards a deeper understanding of the process–(micro)structure–property relationship for open-die forged 7xxx series aluminum alloys regarding their fatigue crack growth resistance.

The main findings are summarized as follows:

1. The damage mechanisms change gradually from a microstructure-sensitive slip plane driven fracture to a microstructure-insensitive multi-slip-system fracture. The FCP rate in the transition zone shows a gradual change of slope until mechanism II is dominant and the Paris regime is initiated.
2. In regime A_2_, we found a correlation between the mean Schmid factor and FCP rate. In detail, an increased Schmid factor from m = 0.413 to m = 0.445 comes with a ~60% higher mean FCP rate.
3. Regime B is independent of the grain structure for L-T-oriented specimens. On the other hand, the smaller grain boundary distances in var 3 (~60% smaller) increase the FCP rate by ~60% in the T-L orientation. This correlation is complemented by the result that a higher aspect ratio L:T correlates with a higher amount of primary phase Al_7_Cu_2_Fe on the fracture surfaces (T-L orientation).
4. Regime C has similar correlations as stage B; however, Al_7_Cu_2_Fe particle clusters and weaker high angle grain boundaries perpendicular to the primary crack can enable crack branching and, consequently, promote a reduction in stress intensity at the primary crack tip.

## Figures and Tables

**Table 1:** Nominal composition of the investigated AA7010-T7452 alloy [wt.%].

Title 1	Zn	Mg	Cu	Fe	Si	Zr
reminder	5.7–6.7	2.1–2.6	1.5–2.0	≤0.15	≤0.12	0.10–0.16

**Table 2:** Estimation of aspect ratio of forged AA7010-T7452 variants var 1–var 4.

Variants	L	T	S
var 1 low deformation	6.2	2.4	1
var 2 medium deformation	14	2.4	1
var 3 large deformation	22	1	1
var 4 alternative casting direction	57	7.8	1

## Data Availability

The data are not publicly available due to data privacy.
